# Supplementary material for: An unusual tandem kinase fusion protein confers leaf rust resistance in wheat
Source: Nat Genet. 2023 May 22;55(6):914–20. doi: 10.1038/s41588-023-01401-2 (PMC10260399; doi:10.1038/s41588-023-01401-2)
Supplement: Supplementary file 1 — Supplementary Notes 1 and 2 and Supplementary Figs. 1–9. [file 41588_2023_1401_MOESM1_ESM.pdf]

nature genetics

Letter

https://doi.org/10.1038/s41588-023-01401-2

An unusual tandem kinase fusion protein confers leaf rust resistance in wheat

In the format provided by the authors and unedited

Table of Contents

Supplementary Note 1 – MutIsoSeq.

Supplementary Note 2 - *Lr9* and *Lr58* are from the same original introgression.

Supplementary Figure 1 - Flow chart illustrating the different steps of MutIsoSeq.

Supplementary Figure 2 - Reports obtained form the *Lr9* (a) and *Lr58* (b) MutIsoSeq runs**.**

Supplementary Figure 3 - The main transcripts of *Lr9* and *Lr58* showed 100% coding sequence (CDS) identity.

Supplementary Figure 4 - Neighbor-joining tree including putative kinase and pseudokinase domains of plant tandem kinase proteins, tandem kinase-vWA proteins, and kinase-vWA proteins.

Supplementary Figure 5 - Neighbor-joining tree constructed using vWA domains of tandem kinase-vWA, kinase-vWA and vWA-containing proteins.

Supplementary Figure 6 – Allele mining using an *Lr9*-specific KASP marker identified one *Ae. umbellulata* accession (TA1851) that might carry *Lr9.*

Supplementary Figure 7 - Linkage between *Lr9* and *Lr58*.

Supplementary Figure 8 - Fluorescence *in situ* hybridization (FISH) using a probe derived from *WTK6-vWA*.

Supplementary Figure 9 - Flow cytometric chromosome analysis and sorting in wheat line TA5605.

Supplementary Note 1

MutIsoSeq

The idea of MutIsoSeq is to use a whole-transcriptome-based approach to rapidly identify candidate transcripts following ethyl methanesulfonate (EMS)-induced mutagenesis. EMS induces point mutations (preferentially G/C to A/T transitions) at random positions across the genome, which can be used to identify candidate genes in forward genetic screens. For the cloning of disease resistance genes, a resistant wild-type parent (Thatcher*Lr9* and TA5605 in this example) is treated with EMS. The resulting progeny (typically a few thousand M2 families in wheat) are phenotypically evaluated for loss of resistance. The assumption is that M2 plants that lost the disease resistance carry a mutation in the disease resistance gene or in a downstream signaling component required for the resistance. Polyploid wheat is particularly suitable for EMS-based disease resistance gene cloning. Because of the genetic redundancy, the majority of the EMS-induced loss-of-resistance mutations are located in the disease resistance gene itself and not in downstream signaling components. In the current example, only one out of 121 loss-of-function mutants did not carry a mutation in *Lr9*. We treated ~2,000 Thatcher*Lr9* grains and ~11,000 TA5605 grains with EMS, which resulted in the identification of 17 and 104 validated susceptible M2 mutant families in the Thatcher*Lr9* and TA5605 genetic backgrounds, respectively. Several rapid-gene cloning protocols have been developed in wheat that combine EMS mutagenesis, genome complexity reduction, and genomics to identify candidate genes[1-4]. MutIsoSeq is a whole transcriptome approach that does not require prior genetic mapping information (Fig. 1). Below, we will exemplify the MutIsoSeq protocol based on the *Lr9/Lr58* cloning.

1) Full-length transcripts of resistant wild-type parent: Full-length transcripts from the resistant wild-type (non-mutagenized) parents Thatcher*Lr9* and TA5605 were generated using PacBio isoform sequencing (Iso-seq). In order to maximize the probability that the causal gene is expressed, RNA was isolated from infected leaf tissue using an avirulent *P. triticina* isolate. RNA samples from four different time points (48, 72, 96, and 120 hours post inoculation) were multiplexed. These time points represent the period during which *P. triticina* invades the leaf and establishes infection. One PacBio SMRT cell was sequenced for each parent (see methods). In total, we obtained 14.2 and 13.5 Gb of raw HiFi data for Thatcher*Lr9* and TA5605, respectively. After data processing (see methods), we obtained 300,066 and 283,330 high-quality transcripts with a mean transcript length of 2,834 bp and 2,849 bp for Thatcher*Lr9* and TA5605, respectively. Iso-seq produces multiple isoforms belonging to the same gene. For MutIsoSeq, it is not necessary to assign transcripts to annotated genes.
2) RNA-seq of loss-of-function mutants: RNA from ten susceptible mutants that lost the *Lr9* and *Lr58*-mediated disease resistances, respectively, was isolated following the same approach as for the Iso-seq. RNA-seq reads were generated using Illumina paired-end sequencing. The aim was to produce around 70 Gb of data for each mutant.
3) The MutIsoSeq pipeline: The RNA-seq reads from the susceptible loss-of-function mutants were mapped against the full-length transcripts of the respective wild-type parent. Single nucleotide polymorphisms (SNPs) were called against the wild-type full-length transcripts. Because the Iso-seq and RNA-seq reads were produced from the same wheat genotype, all SNPs should represent EMS-induced point mutations. For each full-length transcript, the MutIsoSeq pipeline counts the number of SNPs across all mutants. The expectation is that the causal transcript (= the disease resistance gene) will carry non-synonymous EMS-induced point mutations in each of the loss-of-

function mutants. Multiple independent mutants are required in order to distinguish causal point mutations from random background mutations. Polyploid wheat tolerates a relatively high mutation density, ranging from 20 to 42 SNPs per megabase[5]. In total, 187,116 and 145,427 full-length transcripts of Thatcher*Lr9* and TA5605 were retained after processing (reads mapped to the longest isoform and read mapping coverage >5; see methods, Table 1). Considering a GC mutation frequency of $2.43 \cdot 10^{-5}$ (ref. 1), an average GC content of 44.1% (ref. 1) and a mean mRNA length of 2,834 bp, the probability of finding an EMS-induced G/C to A/T transition in a single bread wheat transcript is $(2{,}834 \text{ bp} \cdot 0.441) \cdot 2.43 \cdot 10^{-5} = 0.03$ (see ref. 1 for additional details about calculations). The probability of the same transcript showing independent mutations across *n* number of mutants as a result of chance alone is $(0.03)^n$. Hence, the probability of identifying a false positive candidate transcript rapidly decreases with an increasing number of independent mutants. The following table lists the expected number of transcripts to be mutated simultaneously across a given number of independent mutants:

	**Thatcher*Lr9***	**TA5065**
Total number of transcripts after processing	187,116	145,427
Expected number of transcripts with no mutation	181,255	140,872
Expected number of transcripts to be mutated across 1 mutant	5,683	4,417
Expected number of transcripts to be mutated simultaneously across 2 mutants	173	134
Expected number of transcripts to be mutated simultaneously across 3 mutants	5	4
Expected number of transcripts to be mutated simultaneously across 4 mutants	0.16	0.12
Expected number of transcripts to be mutated simultaneously across 5 mutants	0.0048	0.0036
Expected number of transcripts to be mutated simultaneously across 6 mutants	0.00015	0.00011
Expected number of transcripts to be mutated simultaneously across 7 mutants	4.46E-06	3.47E-06
Expected number of transcripts to be mutated simultaneously across 8 mutants	1.35E-07	1.05E-07
Expected number of transcripts to be mutated simultaneously across 9 mutants	4.11E-09	3.20E-09
Expected number of transcripts to be mutated simultaneously across 10 mutants	1.25E-10	9.71E-11

The table shows that with 4-5 independent mutants, the probability of identifying a false positive transcript becomes very low. With 10 independent mutants, the probability of identifying a false positive transcript that carries 10 EMS-type point mutations as a result of chance alone is $6.7 \cdot 10^{-16}$. The observed number of transcripts that were mutated across *n* mutants is shown in Table 1 and correlates with the expected numbers. The technical details about read processing and mapping are described in the methods section.

Supplementary Note 2

Lr9 and *Lr58* are from the same original introgression

Several lines of evidence indicate that *Lr9* and *Lr58* are from the same original introgression and that *Ae. triuncialis* accession TA10438 is not the donor of *Lr58*:

1) Previously published *Lr9* and *Lr58* markers showed complete linkage with *WTK6-vWA* in the two mapping populations (Extended Data Figs. 3, 4)[6,7].
2) Complementation tests using an F2 population from a Thatcher*Lr9* × TA5605 (133 F2 plants) cross had no susceptible individuals (Supplementary Fig. 7), supporting the linkage of *Lr9* and *Lr58*.
3) Fluorescence *in situ* hybridization using a probe derived from *WTK6-vWA* hybridized to chromosome arm 6BL in both Transfer (*Lr9*) and TA5605 (*Lr58*) (Supplementary Fig. 8). Sequencing of the 3.7-kb probe revealed 100% sequence identity between Thatcher*Lr9* and TA5605 but one polymorphism in the putative *Ae. triuncialis Lr58* donor TA10438.

Together, these results indicated that *Lr9* and *Lr58* are identical and that *Ae. triuncialis* accession TA10438 is not the *Lr58* donor. As the *Lr9* translocation was produced 51 years before the *Lr58* introgression, *Lr58* is likely *Lr9*. During the generation of TA5605, leaf rust-resistant plants with normal chromosome numbers were selected, assuming homoeologous recombination between WL711 (the recurrent bread wheat parent) and *Ae. triuncialis* chromosomes[8]. A rare seed contamination containing *Lr9* may have been selected at this step. Alternatively, materials might have been mislabeled at a later date. The TA5605 and TA10438 accessions used here were the original sources, which were deposited in the Wheat Genetics Resource Center[8]. The reports placing *Lr9* and *Lr58* on different chromosomes (*Lr9* on chromosome 6BL and *Lr58* on chromosome arm 2BL) may be explained by the non-homologous *Lr9* translocation following irradiation. *WTK6-vWA* produced the strongest BLAST hits on the long arms of homoeologous group 2 chromosomes in wheat, in agreement with previous reports showing that the *Ae. umbellulata* chromosome 6U segment from which *Lr9* originated is syntenic to wheat group 2 chromosomes[9,10]. Molecular markers derived from the bread wheat group 2 chromosomes might thus map to the *Ae. umbellulata* translocation, while linked markers derived from the bread wheat chromosome that received the translocation would map to chromosome arm 6BL. We failed to identify *WTK6-vWA* in sequences of flow-sorted TA5605 2B chromosomes, providing additional evidence that *Lr58* is not located on chromosome 2B.

Methods: Flow cytometric chromosome analysis, sorting and sequencing of chromosome 2B from TA5605

Suspensions of mitotic metaphase chromosomes were prepared from root tips of bread wheat line TA5605 according to Vrána et al[11]. The procedure involved cell cycle synchronization in root tip meristems of hydroponically grown young seedlings and accumulation of dividing cells at metaphase using hydroxyurea and amiprophos-methyl, respectively. The roots were then fixed mildly by formaldehyde and intact chromosomes were released into 600 µl ice-cold LB01 buffer[12] by mechanical homogenization of 100 root tips. GAA repeats in chromosomes were labelled by fluorescence *in situ* hybridization in suspension (FISHIS) using 5'-FITC-GAA7-FITC-3' oligonucleotide probes (Sigma, Saint Louis, USA)[13] and chromosomal DNA was stained by DAPI (4′,6-diamidino 2-phenylindole) at 2 µg/ml. Chromosome analysis and sorting was done using a FACSAria II SORP flow cytometer and sorter (Becton Dickinson Immunocytometry Systems, San José, USA). Dot plots FITC vs. DAPI fluorescence (bivariate flow karyotypes) were acquired and chromosome 2B was sorted at rates of 20 to 40 particles per second. Around

42,000 copies of chromosome 2B were sorted into a PCR tube containing 40 μl sterile deionized water. In order to estimate chromosome composition of flow-sorted fractions 1,500 to 2,000 chromosomes were sorted into 10 μl drops of PRINS buffer containing 2.5% sucrose[14] onto a microscopic slide. Air-dried chromosomes were labelled by FISH using the probes for the pSc119.2 repeat, Afa family repeat and 45S rDNA. Chromosome identity and contamination by other chromosomes were determined by microscopic observation of at least 100 chromosomes following the karyotype[14]. Chromosome 2B was identified according to weak telomeric and strong interstitial pSc119.2 signals observed on the short and long arms, respectively (Supplementary Fig. 9). The purity in the sorted chromosome 2B was 94.7%. Chromosome fractions were treated with proteinase K and DNA purified. Twenty nanograms (ng) of DNA was fragmented in 20 μl solution using Bioruptor Plus (Diagenode, Denville, USA) five times for 30 s at HIGH setting. Sequencing libraries were prepared using the NEBNext Ultra™ II DNA Library Prep Kit. DNA libraries were sequenced on Illumina NovaSeq6000, which produced about 82 Gb 2×150 bp paired-end reads for TA5605 that were *de novo* assembled using Meraculous (v2.2.6)[15] using default parameters, resulting in a total assembly size of ~727 Mb (101,896 contigs with N50 of 11.6 kb).

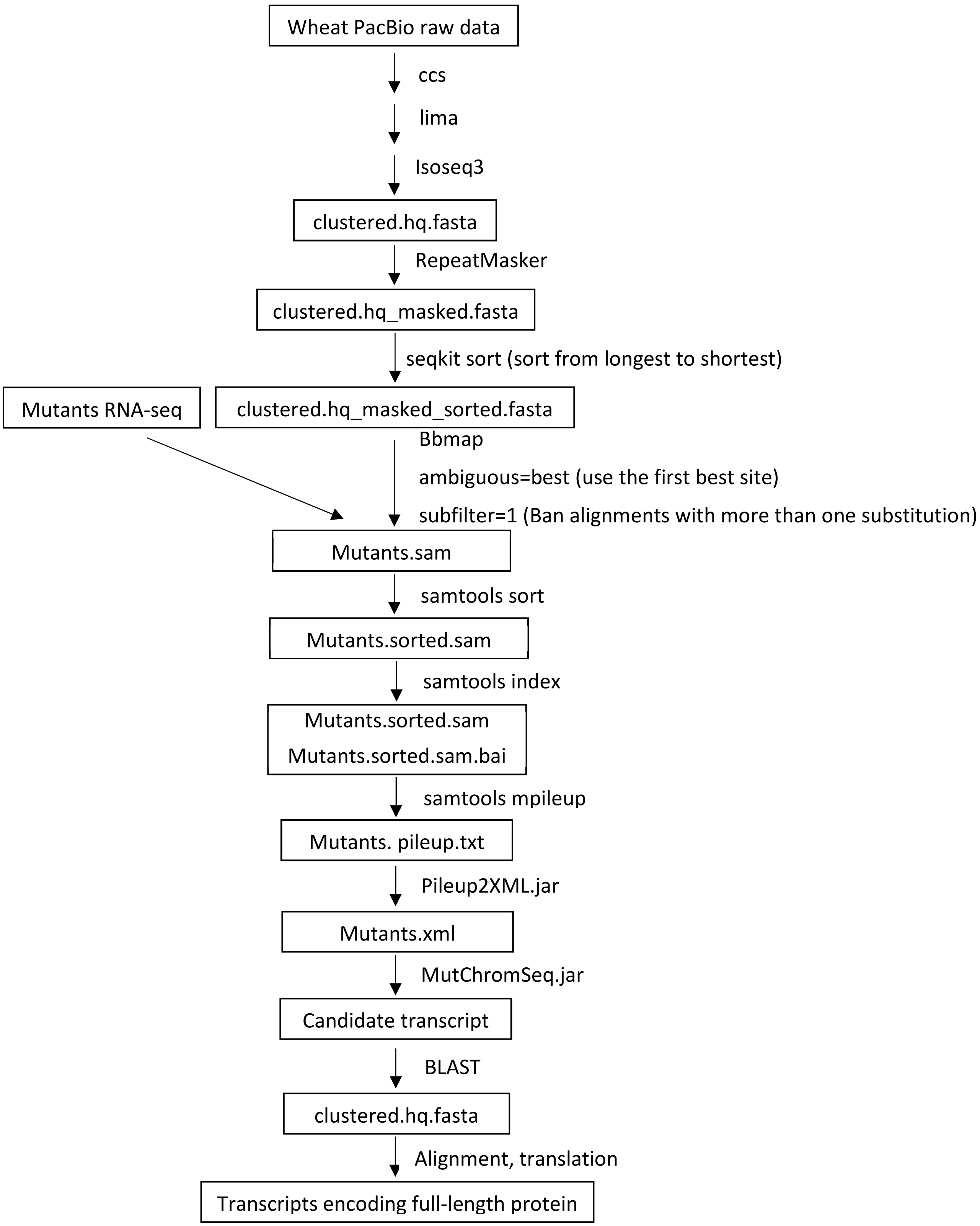

Supplementary Figure 1. Flow chart illustrating the different steps of MutIsoSeq.

a

```
transcript/21507        length:4819
Number Of SNP mutants: 10
Lr9-M812_pileup.txt:    SNP(pos:2338,refallelefreq:0.0,cov:350,C->T)
Lr9-M204_pileup.txt:    SNP(pos:4439,refallelefreq:0.0,cov:295,C->T);SNP(pos:46,refallelefreq:0.0,cov:22,C->T)
Lr9-M369_pileup.txt:    SNP(pos:306,refallelefreq:0.0,cov:104,G->A)
Lr9-M620_pileup.txt:    SNP(pos:632,refallelefreq:0.0,cov:154,G->A)
Lr9-M205_pileup.txt:    SNP(pos:328,refallelefreq:0.0,cov:185,G->A)
Lr9-M13_pileup.txt:     SNP(pos:83,refallelefreq:0.0,cov:32,G->A)
Lr9-M346_pileup.txt:    SNP(pos:484,refallelefreq:0.0,cov:237,G->A)
Lr9-M561_pileup.txt:    SNP(pos:946,refallelefreq:0.0,cov:246,G->A)
Lr9-M279_pileup.txt:    SNP(pos:1050,refallelefreq:0.0,cov:81,C->T);SNP(pos:88,refallelefreq:0.0,cov:15,C->T)
Lr9-M591_pileup.txt:    SNP(pos:2209,refallelefreq:0.0,cov:175,G->A)
```

b

```
transcript/22405        length:4716
Number Of SNP mutants: 10
TA5605-Spt12_pileup.txt:    SNP(pos:525,refallelefreq:0.008928571428571428,cov:224,G->A)
TA5605-Spt10_pileup.txt:    SNP(pos:632,refallelefreq:0.0,cov:198,G->A)
TA5605-Spt20_pileup.txt:    SNP(pos:325,refallelefreq:0.0,cov:72,G->A)
TA5605-Spt7_pileup.txt:     SNP(pos:4246,refallelefreq:0.0,cov:851,G->A)
TA5605-Spt21_pileup.txt:    SNP(pos:4487,refallelefreq:0.0,cov:210,G->A);SNP(pos:2803,refallelefreq:0.002544529262086514,cov:393,G->A)
TA5605-Spt9_pileup.txt:     SNP(pos:801,refallelefreq:0.0,cov:774,G->A)
TA5605-Spt19_pileup.txt:    SNP(pos:3090,refallelefreq:0.0,cov:297,C->T);SNP(pos:510,refallelefreq:0.0,cov:299,C->T)
TA5605-Spt6_pileup.txt:     SNP(pos:4246,refallelefreq:9.049773755656109E-4,cov:1105,G->A)
TA5605-Spt25_pileup.txt:    SNP(pos:376,refallelefreq:0.006535947712418301,cov:153,C->T)
TA5605-Spt1_pileup.txt:     SNP(pos:4307,refallelefreq:0.0,cov:755,G->A)
```

Supplementary Figure 2. Reports obtained form the *Lr9* (a) and *Lr58* (b) MutIsoSeq runs.

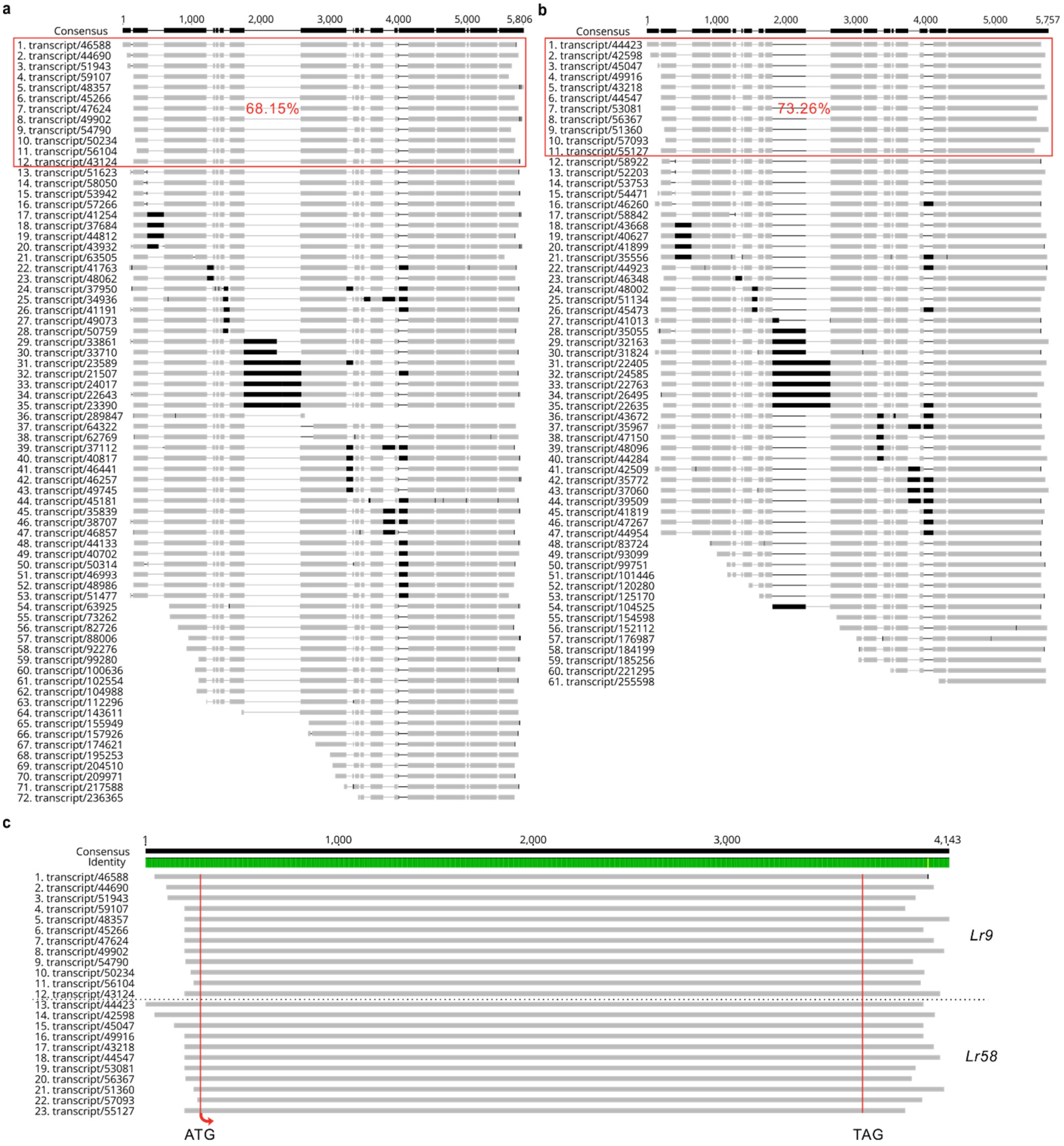

Supplementary Figure 3. The main transcripts of *Lr9* and *Lr58* showed 100% coding sequence (CDS) identity. a, Seventy-two different transcripts were retrieved for the *Lr9* candidate from Thacther*Lr9*. The 12 main transcripts accounted for 68.15% of the total *Lr9* transcripts (calculated using the coverage number of transcripts, representing the number of molecules sequenced in the PacBio SMRT cells). These 12 main transcripts encode the same protein. **b,** Sixty-one different transcripts were retrieved for the *Lr58* candidate from TA5605. The 11 main transcripts accounted for 73.26% of the total *Lr58* transcripts. The 11 main transcripts encode the same protein **c,** The main transcripts of *Lr9* and *Lr58* showed 100% coding sequence identity.

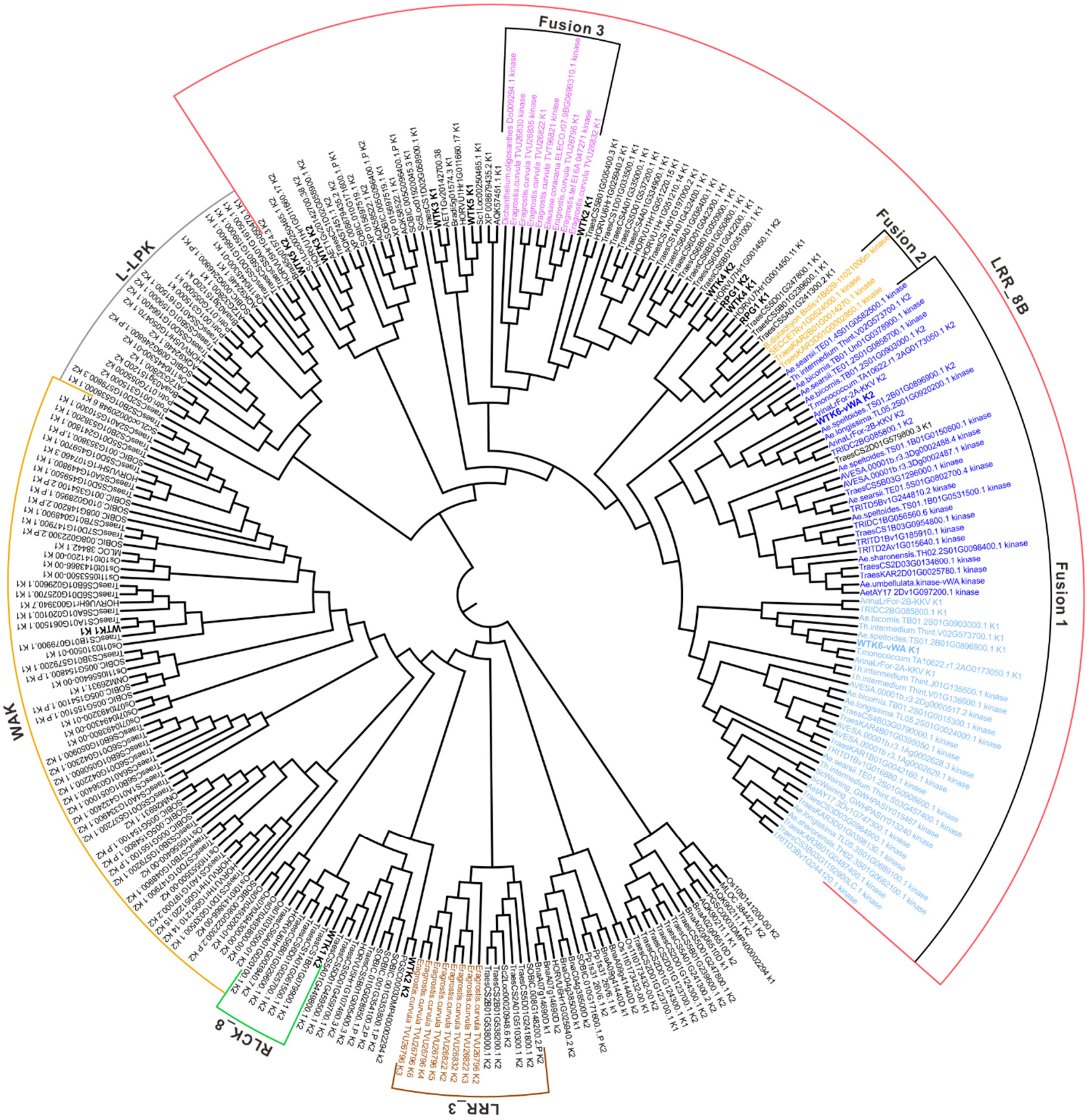

Supplementary Figure 4. Neighbor-joining tree including putative kinase and pseudokinase domains of plant tandem kinase proteins, tandem kinase-vWA proteins, and kinase-vWA proteins. LRR_8B (cysteine-rich kinases), L-LPK (concanavalin A-like lectin protein kinases), WAK (cell wall-associated kinases), RLCK_8 (receptor-like cytoplasmic kinase subfamily 8) and LRR_3 (leucine-rich-repeat receptor kinase subfamily 3)[16]. Kinase domains in blue, orange and purple font colors represent the three independent kinase-vWA fusion events. Light and dark blue font colors represent kinase domains that are orthologous to WTK6-vWA_kinase1 and WTK6-vWA_kinase2, respectively. Kinase domains in brown font color represent C-terminal kinase fusions found in *Eragrostis curvula.* Kinase domains from wheat tandem kinases (WTKs) involved in disease resistance are indicated in bold.

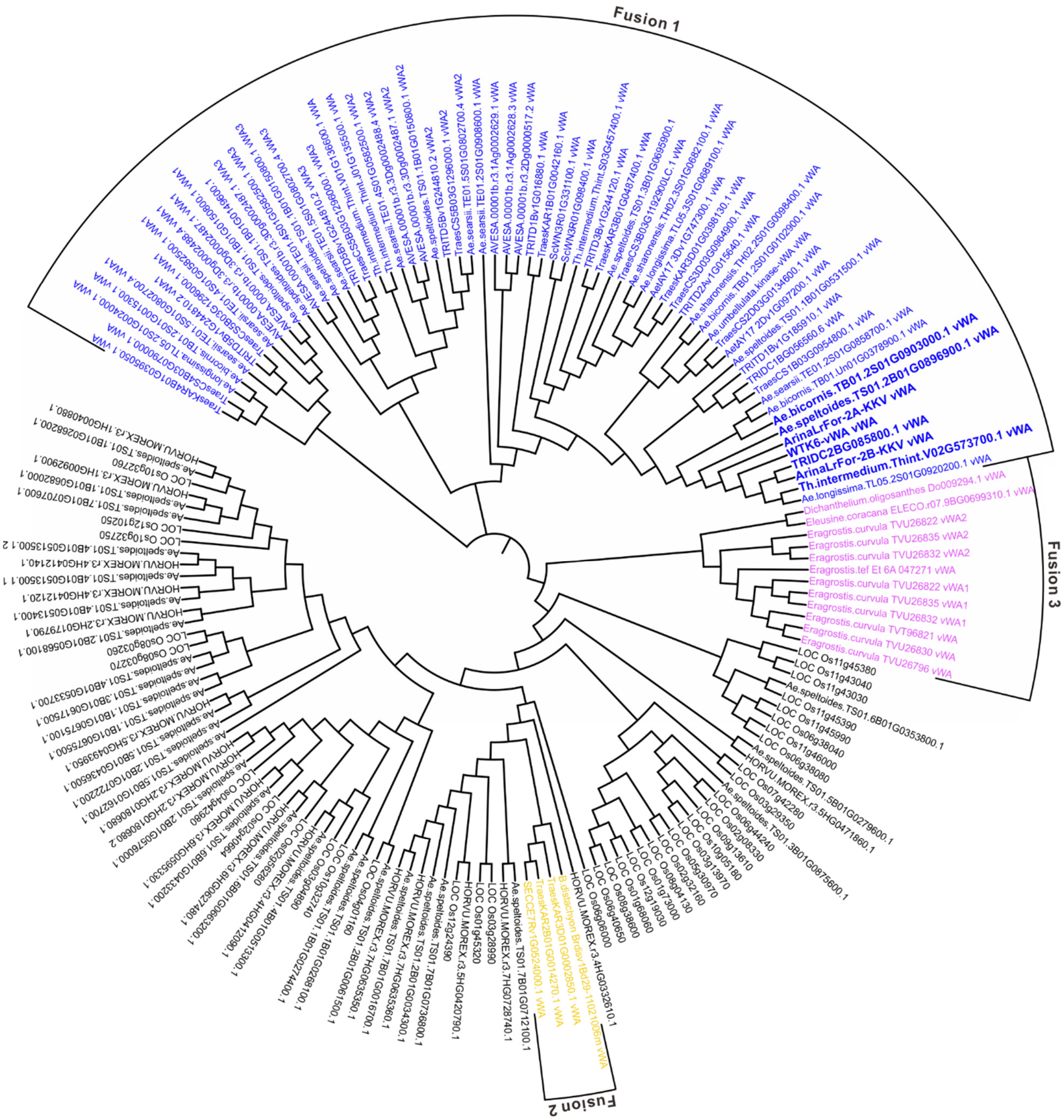

Supplementary Figure 5. Neighbor-joining tree constructed using vWA domains of tandem kinase-vWA, kinase-vWA and vWA-containing proteins. vWA domains in blue, orange and purple font colors represent the three independent kinase vWA gene fusion events. vWA domains in bold represent tandem kinase-vWAs.

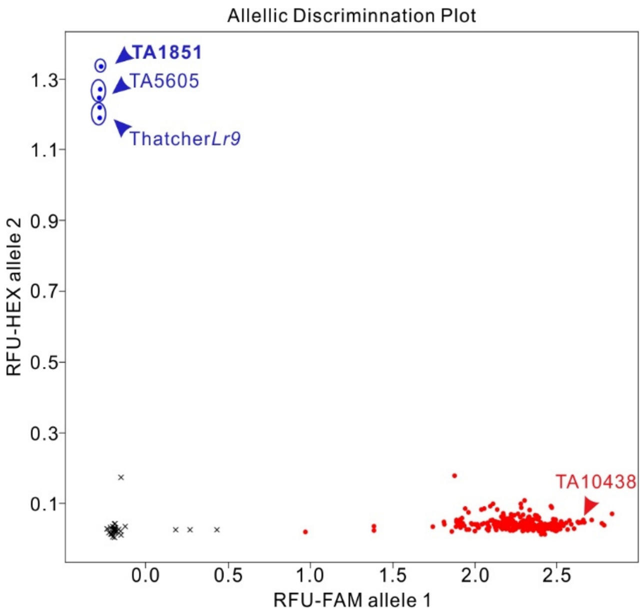

Supplementary Figure 6. Allele mining using an *Lr9*-specific KASP marker identified one *Ae. umbellulata* accession (TA1851) that might carry *Lr9*. The diversity panel included 205 *Ae. triuncialis* and 59 *Ae. umbellulata* accessions. Blue dots represent the *Lr9*-carying controls Thatcher*Lr9* and TA5605, as well as the putative *Lr9* donor TA1851. Red dots correspond to the alternative allele. Black crosses indicate no amplification product.

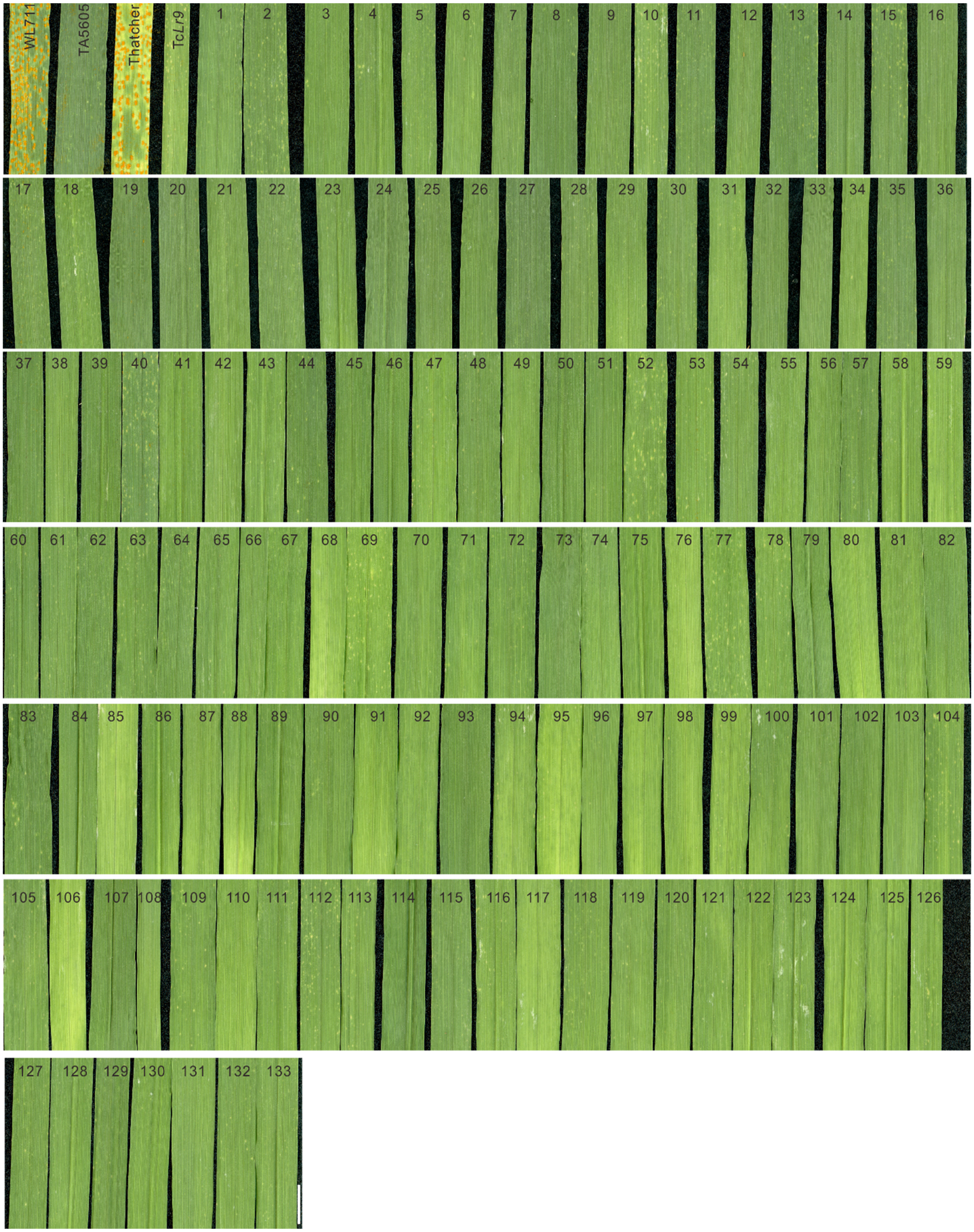

Supplementary Figure 7. Linkage between *Lr9* and *Lr58*. Shown are representative images of an F2 populations derived from a cross between Thatcher*Lr9* and TA5605 inoculated with the avirulent *P. triticina* isolate B9414. Images were taken 12 days after inoculation. Scale bar = 1 cm.

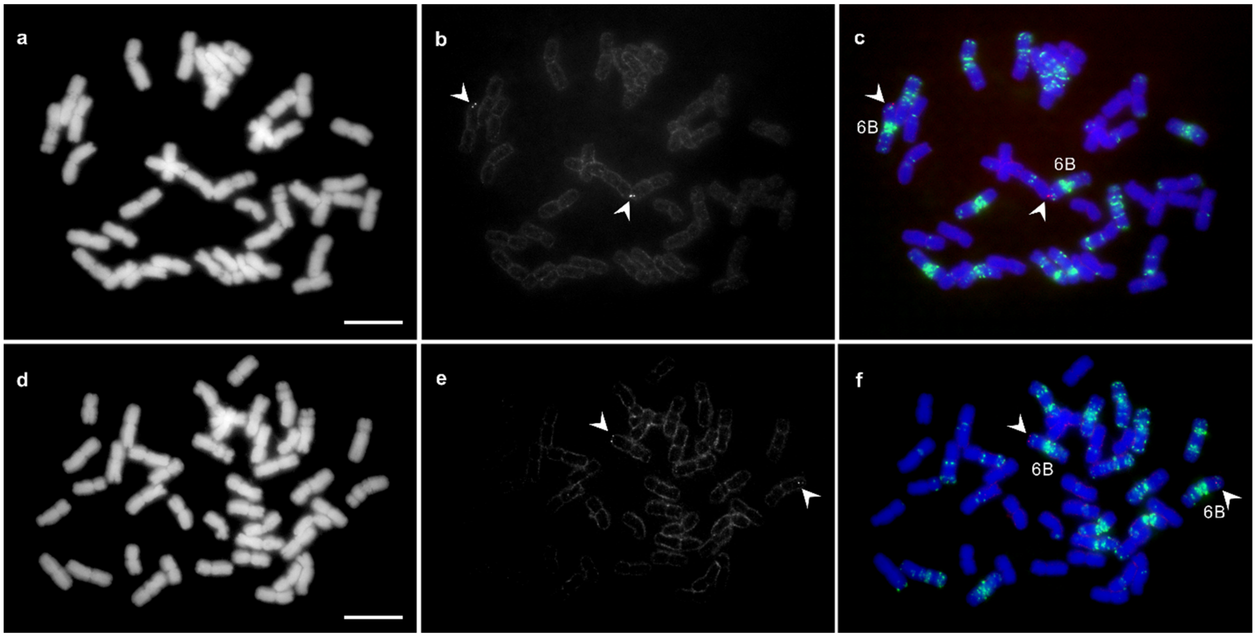

Supplementary Figure 8. Fluorescence *in situ* hybridization (FISH) using a probe derived from *WTK6-vWA*. (**a-c**) TA5524 (Transfer, *Lr9*) and (**d-f**) TA5605 (*Lr58*). The *WTK6-vWA* signal (arrowheads) was detected on the telomeric end of chromosome arm 6BL in both accessions. **a** and **d** show DAPI stained mitotic chromosomes; **b** and **e** show FISH signals (arrowheads); **c** and **f** are merged images. GAA repeats (green signals) were used to identify the homologous chromosome pairs in wheat. Experiment was performed in two different accessions (TA5524, TA5605) with three plants from each accession. Similar FISH patterns were observed in 10 metaphase cells analyzed from each plant in two different accessions. Scale bar = 10 μm.

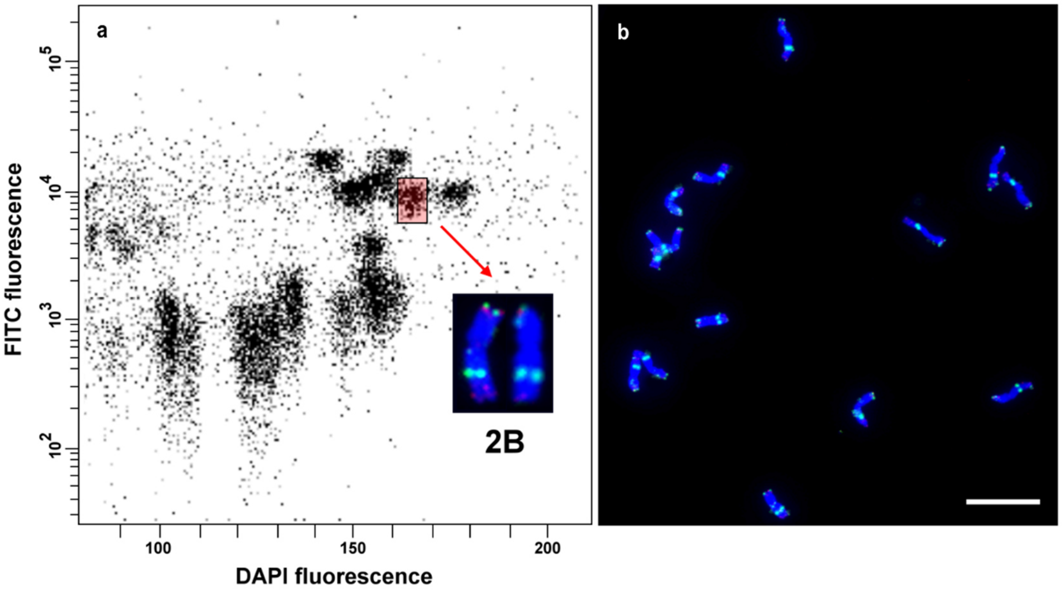

Supplementary Figure 9. Flow cytometric chromosome analysis and sorting in wheat line TA5605. Prior to the analysis, chromosomes in suspensions were labeled by fluorescence *in situ* hybridization in suspension (FISHIS) with FITC-conjugated probes for GAA microsatellites and their DNA was stained by DAPI. **a,** Bivariate flow karyotype FITC fluorescence vs. DAPI fluorescence. Chromosome 2B was sorted using the sort window shown as red rectangle at purities of 94.7%. Inset: images of the flow sorted chromosome 2B. **b,** Example of 2B chromosomes sorted onto a microscope slide after FISH with probes for pSc119.2 repeat (green), Afa family repeat (red) and 45S rDNA (yellow); chromosomes were counterstained with DAPI (blue). The experiment was repeated independently twice with similar results. Scale bar = 10 µm.

References

1. Sanchez-Martin, J. *et al.* Rapid gene isolation in barley and wheat by mutant chromosome sequencing. *Genome Biology* **17**, 221 (2016).
2. Thind, A.K. *et al.* Rapid cloning of genes in hexaploid wheat using cultivar-specific long-range chromosome assembly. *Nature Biotechnology* **35**, 793-796 (2017).
3. Steuernagel, B. *et al.* Rapid cloning of disease-resistance genes in plants using mutagenesis and sequence capture. *Nature Biotechnology* **34**, 652-655 (2016).
4. Arora, S. *et al.* Resistance gene cloning from a wild crop relative by sequence capture and association genetics. *Nature Biotechnology* **37**, 139-143 (2019).
5. Bettgenhaeuser, J. & Krattinger, S.G. Rapid gene cloning in cereals. *Theoretical and Applied Genetics* **132**, 699-711 (2019).
6. Gupta, S.K., Charpe, A., Koul, S., Prabhu, K.V. & Haq, Q.M.R. Development and validation of molecular markers linked to an *Aegilops umbellulata*–derived leaf-rust-resistance gene, *Lr9*, for marker-assisted selection in bread wheat. *Genome* **48**, 823-830 (2005).
7. Kuraparthy, V., Sood, S., Guedira, G.-B. & Gill, B.S. Development of a PCR assay and marker-assisted transfer of leaf rust resistance gene *Lr58* into adapted winter wheats. *Euphytica* **180**, 227-234 (2011).
8. Kuraparthy, V. *et al.* A cryptic wheat-*Aegilops triuncialis* translocation with leaf rust resistance gene *Lr58*. *Crop Science* **47**, 1995-2003 (2007).
9. Friebe, B., Jiang, J., Tuleen, N. & Gill, B.S. Standard karyotype of *Triticum umbellulatum* and the characterization of derived chromosome addition and translocation lines in common wheat. *Theoretical and Applied Genetics* **90**, 150-156 (1995).
10. Said, M. *et al.* Development of DNA markers from physically mapped loci in *Aegilops comosa* and *Aegilops umbellulata* using single-gene FISH and chromosome sequences. *Frontiers in Plant Science* **12**, 689031 (2021).
11. Vrána, J. *et al.* Flow sorting of mitotic chromosomes in common wheat (*Triticum aestivum* L.). *Genetics* **156**, 2033-2041 (2000).
12. Doležel, J., Binarová, P. & Lucretti, S. Analysis of nuclear DNA content in plant cells by flow cytometry. *Biologia Plantarum* **31**, 113-120 (1989).
13. Giorgi, D. *et al.* FISHIS: fluorescence in situ hybridization in suspension and chromosome flow sorting made easy. *PLoS One* **8**, e57994 (2013).
14. Kubaláková, M., Macas, J. & Doležel, J. Mapping of repeated DNA sequences in plant chromosomes by PRINS and C-PRINS. *Theoretical and Applied Genetics* **94**, 758-763 (1997).
15. Chapman, J.A. *et al.* Meraculous: *de novo* genome assembly with short paired-end reads. *PLoS One* **6**, e23501 (2011).
16. Zulawski, M., Schulze, G., Braginets, R., Hartmann, S. & Schulze, W.X. The *Arabidopsis* Kinome: phylogeny and evolutionary insights into functional diversification. *BMC Genomics* **15**, 548 (2014).
