# Supplementary material for: An unusual tandem kinase fusion protein confers leaf rust resistance in wheat
Source: Nat Genet. 2023 May 22;55(6):914–20. doi: 10.1038/s41588-023-01401-2 (PMC10260399; doi:10.1038/s41588-023-01401-2)
Supplement: Supplementary file 2 — Reporting Summary [file 41588_2023_1401_MOESM2_ESM.pdf]

nature portfolio

Corresponding author(s): Simon Krattinger

Last updated by author(s): Mar 19, 2023

Reporting Summary

Nature Portfolio wishes to improve the reproducibility of the work that we publish. This form provides structure for consistency and transparency in reporting. For further information on Nature Portfolio policies, see our Editorial Policies and the Editorial Policy Checklist.

Statistics

For all statistical analyses, confirm that the following items are present in the figure legend, table legend, main text, or Methods section.

n/a	Confirmed	
☐	☒	The exact sample size (*n*) for each experimental group/condition, given as a discrete number and unit of measurement
☒	☐	A statement on whether measurements were taken from distinct samples or whether the same sample was measured repeatedly
☐	☒	The statistical test(s) used AND whether they are one- or two-sided<br>*Only common tests should be described solely by name; describe more complex techniques in the Methods section.*
☒	☐	A description of all covariates tested
☒	☐	A description of any assumptions or corrections, such as tests of normality and adjustment for multiple comparisons
☒	☐	A full description of the statistical parameters including central tendency (e.g. means) or other basic estimates (e.g. regression coefficient) AND variation (e.g. standard deviation) or associated estimates of uncertainty (e.g. confidence intervals)
☐	☒	For null hypothesis testing, the test statistic (e.g. F, t, r) with confidence intervals, effect sizes, degrees of freedom and P value noted<br>*Give P values as exact values whenever suitable.*
☒	☐	For Bayesian analysis, information on the choice of priors and Markov chain Monte Carlo settings
☒	☐	For hierarchical and complex designs, identification of the appropriate level for tests and full reporting of outcomes
☒	☐	Estimates of effect sizes (e.g. Cohen's *d*, Pearson's *r*), indicating how they were calculated

Our web collection on statistics for biologists contains articles on many of the points above.

Software and code

Policy information about availability of computer code

Data collection: All the leaf images were scanned using an Epson Perfection V600 Photo scanner and corresponding software.

Data analysis:

MutIsoSeq:
IsoSeq v3
SeqKit v2.2.0
BBMap v.38.96
Samtools v1.6
Pileup2XML.jar
MutChromSeq.jar
TBtools v1.09873

Protein structure prediction and visualization:
AlphaFold v2.0
PyMOL 2.3.0

Genome assembly and evaluation:
Hifiasm v.0.16.1
QUAST 5.0.2
BBMap v.38.96
Meraculous v2.2.6

Lr9 translocation assembly:
Minimap2 v.2.21
Bedtools v2.30.0
Mosdepth v0.3.3
TBtools v1.09873
Jellyfish v. 2.2.10
BWA mem v.0.7.17
Geneious Prime Version 2020.2.4 and Version 2022.1.1
BBMap v.38.96

Sequence alignment analysis:
Geneious Prime Version 2020.2.4

Phylogenetic analysis:
Clustal Omega (https://www.ebi.ac.uk/Tools/msa/clustalo/)
iTOL (https://itol.embl.de/)

Gene projection:
Liftoff v1.6.3

Figure generation/ Drawing:
CorelDRAW 2018
BioRender (https://www.biorender.com/)

For manuscripts utilizing custom algorithms or software that are central to the research but not yet described in published literature, software must be made available to editors and reviewers. We strongly encourage code deposition in a community repository (e.g. GitHub). See the Nature Portfolio guidelines for submitting code & software for further information.

Data

Policy information about availability of data

All manuscripts must include a data availability statement. This statement should provide the following information, where applicable:

- Accession codes, unique identifiers, or web links for publicly available datasets
- A description of any restrictions on data availability
- For clinical datasets or third party data, please ensure that the statement adheres to our policy

Data supporting the findings of this work are available within the paper and its Supplementary Information.

The raw Iso-seq and RNA-seq data used for MutIsoSeq, the PacBio CCS reads used for de novo whole-genome assemblies, the Illumina raw reads of TA5605, TA10438, Thatcher and sorted TA5605 chromosome 2B were deposited in the European Nucleotide Archive (ENA) under study number PRJEB53839.

The Lr9 genomic and mRNA sequences were deposited in NCBI Genbank under accession numbers ON872164 and ON872165.

The genome assemblies of ThatcherLr9 and Aegilops umbellulata accessionTA1851, the assembly of sorted TA5605 chromosome 2B, the clustered Iso-seq transcripts of ThatcherLr9 and TA5605 and the CDS and genomic sequence of Lr9 are available on the DRYAD database under https://doi.org/doi:10.5061/dryad.gxd2547pw.

The following public databases were used in the study:

whole-genome assemblies of 12 bread wheat cultivars, durum wheat, wild emmer wheat, and six Aegilops species
WheatOmics 1.0 (http://202.194.139.32/blast/blast.html)

whole-genome assemblies of Rye (Lo7 and Weining), hexaploid oat (OT3098) and barley (Morex)
GrainGenes (https://wheat.pw.usda.gov/blast/)

whole-genome assemblies of Thinopyrum intermedium, Brachypodium distachyon, Dichanthelium oligosanthes and Eleusine coracana
Phytozome (https://phytozome-next.jgi.doe.gov)

whole-genome assemblies of Eragrostis tef and Eragrostis curvula
GoGe (https://genomevolution.org/coge/)

Human research participants

Policy information about studies involving human research participants and Sex and Gender in Research.

Reporting on sex and gender	N/A
Population characteristics	N/A
Recruitment	N/A

Ethics oversight | N/A

Note that full information on the approval of the study protocol must also be provided in the manuscript.

Field-specific reporting

Please select the one below that is the best fit for your research. If you are not sure, read the appropriate sections before making your selection.

☒ Life sciences ☐ Behavioural & social sciences ☐ Ecological, evolutionary & environmental sciences

For a reference copy of the document with all sections, see nature.com/documents/nr-reporting-summary-flat.pdf

Life sciences study design

All studies must disclose on these points even when the disclosure is negative.

Sample size	Sample sizes are described in the manuscript. Sizes of ThatcherLr9 x Avocet S, TA5605 x Avocet S and ThatcherLr9 x TA5605 F2 populations are 136, 128 and 133 plants, representing 272, 256, and 266 gametes, respectively. The sizes of the mapping populations were based on literature and based on the calculated recombination frequency (the genetic resolution of a mapping population with 250 gametes is 0.4 cM, which is sufficient to demonstrate linkage between Lr9/Lr58 and a molecular marker derived from the candidate gene). Size of ThatcherLr9 mutant population = 919, size of TA5605 mutant population = ~7,400. We screened an extremely large mutant population to obtain a high number of susceptible mutants with mutations in Lr9, which has been used for protein domain functional interpretation. The population size is bigger than most reported population sizes in similar studies. No statistical method was used to predetermine sample size.
Data exclusions	No data were excluded.
Replication	The 17 ThacthterLr9 mutants were phenotyped twice at the M2 generation and validated twice at M3 generation. The TA5605-Spt1 to Spt123 mutants were phenotyped once at M2 generation, and validated at least once at M3 generation. The TA5605-Spt124 to 186 mutants were phenotyped twice at M2 generation. All segregating F2 populations were phenotyped and genotyped once. The silencing experiment was performed independently three times with at least five biological replicates per experiment, which all gave similar results. The genomic in situ hybridization experiment was was performed independently twice with with similar results. RT-qPCR analysis of WTK6-vWA was applied once with three biological replicates, and standard deviations were calculated based on values of replicates.
Randomization	Inoculated plants were allocated randomly among groups.
Blinding	Blinding was performed when phenotyping plants (i.e., the genotype of the plant was not known when the phenotypic data were recorded).

Reporting for specific materials, systems and methods

We require information from authors about some types of materials, experimental systems and methods used in many studies. Here, indicate whether each material, system or method listed is relevant to your study. If you are not sure if a list item applies to your research, read the appropriate section before selecting a response.

Materials & experimental systems

n/a	Involved in the study	
☒	☐	Antibodies
☒	☐	Eukaryotic cell lines
☒	☐	Palaeontology and archaeology
☒	☐	Animals and other organisms
☒	☐	Clinical data
☒	☐	Dual use research of concern

Methods

n/a	Involved in the study	
☒	☐	ChIP-seq
☒	☐	Flow cytometry
☒	☐	MRI-based neuroimaging
